# Ticket to perform: an explorative study of trainees’ engagement in and transfer of surgical training

**DOI:** 10.1186/s12909-023-04048-z

**Published:** 2023-01-26

**Authors:** Sigurd Beier Sloth, Rune Dall Jensen, Mikkel Seyer-Hansen, Gunter De Win, Mette Krogh Christensen

**Affiliations:** 1 Centre for Educational Development, Aarhus University, Trøjborgvej 82-84, 8200 Aarhus, Denmark; 2 Department of Clinical Medicine, Aarhus University, Palle Juul-Jensens Boulevard 11, 8200 Aarhus, Denmark; 3 Corporate HR, MidtSim, Central Denmark Region, Palle Juul-Jensens Boulevard 82, 8200 Aarhus, Denmark; 4 Department of Gynaecology and Obstetrics, Aarhus University Hospital, Palle Juul-Jensens Boulevard 99, 8200 Aarhus, Denmark; 5 Antwerp Surgical Training, Anatomy and Research Center (ASTARC), Faculty of Medicine and Health Sciences, University of Antwerp, Universiteitsplein 1, 2610 Wilrijk, Belgium; 6 Department of Urology, University Hospital Antwerp, Drie Eikenstraat 655, 2650 Edegem, Belgium

**Keywords:** Surgical training, Simulation, Transfer of training, Self-regulated learning, Laparoscopy

## Abstract

**Background:**

Research suggests that simulation-based surgical skills training translates into improved operating room performance. Previous studies have predominantly focused on training methods and design and subsequent assessable performances and outcomes in the operating room, which only covers some aspects of training engagement and transfer of training. The purpose of this qualitative study was to contribute to the existing body of literature by exploring characteristics of first-year trainees’ engagement in and perceptions of transfer of surgical skills training.

**Methods:**

We conducted an explorative study based on individual interviews with first-year trainees in General Surgery, Urology, and Gynaecology and Obstetrics who participated in a laparoscopic skills training program. Informants were interviewed during and two months after the training program. A thematic cross-case analysis was conducted using systematic text condensation.

**Results:**

We interviewed 12 informants, which produced 24 transcripts for analysis. We identified four main themes: (1) sportification of training, (2) modes of orientation, (3) transferrable skills, and (4) transfer opportunities. Informants described their surgical training using sports analogies of competition, timing, and step-by-step approaches. Visual orientations, kinaesthetic experiences, and elicited dialogues characterised training processes and engagement. These characteristics were identified in both the simulated and the clinical environment. Experiences of specific skills transfer included ambidexterity, coordination, instrument handling, and visuospatial ability. General transfer experiences were salient in informants’ altered training approaches. Informants considered the simulation-based training an entry ticket to perform in the operating room and mentioned supervisor-trainee relationships and opportunities in the workplace as critical conditions of transfer.

**Conclusions:**

Our findings elucidate characteristics of surgical training engagement that can be interpreted as self-regulated learning processes that transcend surgical training environments. Despite appreciating the immediate skills improvements resulting from training, trainees’ narratives reflected a struggle to transfer their training to the clinical setting. Tensions existed between perceptions of transferable skills and experiences of transfer within the clinical work environments. These results resonate with research emphasising the importance of the work environment in the transfer process. Our findings provide insights that may inform the development of training programs that support self-regulated learning and transfer of training from the simulated to the clinical environment.

## Background

Becoming an expert surgeon entails pursuing a challenging and continuous postgraduate surgical training path. In addition to acquiring expert knowledge in diagnostics and treatments, surgical trainees must develop a high level of technical and non-technical skills. Simulation-based surgical skills training (SBST) has proven effective for developing trainees’ surgical skills [1]. Research has strengthened the evidence of skills transfer to the operating room (OR), reducing adverse events and increasing patient safety [2, 3]. However, previous literature emphasises a more holistic view of the transfer of training processes, consisting of an interplay of trainee characteristics, training design, and the work environment [4–6].

Different theoretical and conceptual frameworks have been developed and applied to explain learning and training processes in attaining expertise in surgery. Ericsson’s theoretical framework of deliberate practice [7, 8] has largely inspired the development of modern SBST designs, including proficiency-based training (PBT). PBT is a goal-oriented training approach that allows time for deliberate practice, accommodates individual progression pace and ensures that all trainees reach minimum performance standards [9]. PBT has proven effective for laparoscopic skills training, resulting in improved OR performances and skills retention [3, 10, 11].

Self-regulated learning (SRL) theories have been applied and investigated in medical education and simulation-based training [12–14]. While different theoretical frameworks exist, what they have in common is the idea that SRL is a dynamic process where the learner modulates learning experiences and adapts learning strategies to reach desired learning goals [15–18]. However, research on SRL in SBST is sparse. As pointed out by Brydges et al. (2015), SRL has largely been misinterpreted as self-learning, depicting the trainee as exclusively responsible for learning [13]. By contrast, as stated by Pintrich (1999), “self-regulated learning is neither easy nor automatic” [16]. SRL involves strategies that the learner uses and develops to regulate cognitive, affective, behavioural processes and resources throughout a learning activity. As such, SRL must be recognised as a complex and dynamic system of self-regulatory processes that should be assisted by evidence-based educational supports, especially if the goal is to promote ongoing SRL in postgraduate training [13, 19]. Previous research has demonstrated that trainees who engage in self-regulatory activities tend to learn more than those who struggle to self-regulate [17].

While previous studies have mainly focused on different structures and designs for SBST and assessing immediate and delayed transfer outcomes in the OR [2], less attention has been given to how trainees engage in and regulate their surgical training and how they transfer their training to the clinical environment. It is reasonable that much attention has concentrated on proving that SBST leads to improved OR performances. However, to advance the knowledge about trainees’ actual conditions for transfer of training from the simulated learning environment to the clinical setting, we need in-depth studies on how trainees engage in training and their perceived conditions for transfer of training. A study by Blackhall et al. (2018) explored facilitators and barriers towards engagement in home-based SBST [20]. Among key findings were that trainees did not perceive a connection between training tasks and their clinical practice and that trainees lacked motivation to engage in training because they were more focused on activities leading to career progression than on the development of their surgical skills. Moreover, engagement in simulation-based training has been shown to be complex and influenced by social and cultural factors [20–22].

Building on previous research, we wanted to further explore this apparent disconnect in the transfer of training by conducting in-depth interviews on how surgical trainees perceive the transfer of their training into clinical practice. Furthermore, rather than focusing on structural and psychological barriers and facilitators of training engagement, this study aimed to explore characteristics of learning processes that take place when trainees engage in surgical training. With these aims in mind, our objective was to contribute with new perspectives that may inform the development of educational interventions that support SRL and transfer of training within postgraduate surgical training.

## Methods

We conducted a qualitative study using a constructivist research approach. Interviews provided insights into informants’ experiences, acknowledging that meanings, experiences and interpretations are constructed iteratively among informants, interviewers and researchers [23].

### Context and sample

Informants were regularly invited on a voluntary basis among trainees participating in a six-week simulation-based laparoscopic skills training program. The training was situated and facilitated in two ways, i.e., by centralised or remote training, based on a previously published randomised comparative study investigating training patterns and performance outcomes between the two training modalities [24]. The training was structured as PBT, and training equipment was the same between groups. In comparison, the remote training group trained on their own, guided by thorough written and visual instructions and provided short written feedback on submitted task videos, while the centralised group trained for two full days with instructors present to instruct and give verbal feedback. In the previous study, we found that remote training facilitated distributed practice and that the two training modalities resulted in comparable performance outcomes [24].

Rather than a comparative research approach, the present study focused on exploring characteristic processes of engagement in surgical training and on trainees’ perceptions of transfer of training, irrespective of training mode. The study population was Danish first-year trainees in General Surgery, Urology, and Gynaecology and Obstetrics who had not previously participated in a postgraduate laparoscopic skills training program. As part of the informant selection process, a purposeful heterogeneity sampling strategy was used, which is a selection method that seeks to represent the study population as widely as possible to achieve a holistic understanding of the phenomena under investigation [25].

### Data collection

From September 2019 to February 2020, informants participated in two individual interviews, each lasting 40 to 60 minutes. We conducted the first interview in the middle of the six-week training program and the second approximately two months after the program had concluded. We did this to allow time for informants to build perceptions and experiences and to capture perspectives of transfer of training both during and after the training programme. Time between interviews allow additional reflection leading to an increased level of detail in the narrative data [26, 27].

Two experienced qualitative researchers conducted the interviews using a semi-structured interview guide. We designed the interview guide with suggested open-ended questions on how trainees engaged in training and how they experienced conditions for applying trained skills into their clinical practice. The interview guide was informed by Zimmerman’s (2000) theoretical frameworks of SRL [15] and Grossman and Salas’(2011) adapted model of the transfer process [6]. Examples of questions were, “describe an optimal training situation” and “how do you see the connections between the simulation-based training and your clinical training?”. Meaning saturation was assessed continuously and reached by conducting follow-up interviews and focusing on meaning units instead of codes [28]. Interviews were audio-recorded and transcribed verbatim.

### Data analysis

We conducted a thematic cross-case analysis using systematic text condensation (STC) [29]. STC includes four analytical steps. In step 1, we formed an overall impression of the data, identifying preliminary themes that served as the starting point for further analyses. In step 2, we reviewed all transcripts identifying meaning units (i.e., text fragments containing information about the research questions). Using code labels, we connected related meaning units into four thematic code groups. We repeatedly met and discussed identified meaning units and corresponding code groups. In step 3, we sorted the meaning units of each code group into subgroups. Amalgamating the content from meaning units, we reduced each subgroup into a text condensate, maintaining expressions originally used by the informants (Tables 2, 3, 4 and 5). In step 4, we interpreted and reconceptualised the data, developing descriptions elucidating the research questions. Using constant comparison, we ensured that the synthesised descriptions and concepts still reflected the validity and wholeness of the original interviews. We chose STC because it offers a transparent and collaborative approach to thematic cross-case analysis where text condensations elucidate what led to the syntheses [29]. The variety of backgrounds represented in the research group provided an opportunity to analyse and interpret emergent themes from different viewpoints and perspectives [30].

## Results

Twelve informants were interviewed (Table 1) resulting in a total of 24 transcripts for analysis.

**Table 1:** Informant characteristics

**Sex**	
Male (n)	4
Female (n)	8
**Age**	
Median (range)	29 (27–35)
**Postgraduate years**	
Median (range)	1.5 (1–5)
**Specialty**	
General Surgery (n)	6
Gynaecology and Obstetrics (n)	4
Urology (n)	2
**Training group**	
Centralised training (n)	6
Remote training (n)	6

We identified four main themes: (1) sportification of training, (2) modes of orientation, (3) transferrable skills and (4) transfer opportunities. Our results represent overall perspectives and conceptualisations of the informants’ narrated experiences. Accompanying text condensates (Tables 2, 3, 4 and 5) illustrate multivocal narratives within the subgroups of each theme (step 3 of the analysis).

**Table 2:** Text condensates for subgroups within the theme ‘sportification of training’

**Subgroup**	**Text condensate**
**Competition**	Training has become like a sport for me, and I sometimes have a hard time limiting myself. It is probably my competitive nature. I have a hard time letting go of the training which can also be a bit challenging sometimes. It is primarily a competition with myself. However, I cannot help comparing myself with my peers. I have an inner drive to be skilled. I find all kinds of competition fun as long as the level is not out of my reach. So, competition is very motivating for me *Constructed from 7 different informants’ expressions*
**Timing**	I want to be in the best category to prove that I am capable. It is not like it is a complete tragedy if I do not obtain the best time and grades, but that is the goal. I started by writing down the task times, trying to get faster. The timing of tasks means a lot to me, primarily because it is decisive for the task grading and because I know that when I can do it fast and fluently, it usually means I am proficient *Constructed from 6 different informants’ expressions*
**Step-by-step approach**	I think the optimal way for me is to structure the training in steps: ‘First, train this step—repeat it—master it—add the next step—train it—master it.’ I gain more successful experiences that way instead of taking on the entire task right away. I separate the tasks and procedures into steps so it becomes more manageable. I try to develop systems and say to myself, ‘first that, then that, and then that’—small steps at a time that I can train without too much pressure and get feedback afterwards. When I master each step, and the transitions between the steps run smoothly, that is when I perform well *Constructed from 7 different informants’ expressions*

**Table 3:** Text condensates for subgroups within the theme ‘modes of orientation’

**Subgroup**	**Text condensate**
**Visual**	I go through the task in my head, almost like a movie. I imagine that it is my own movements in that movie. If I cannot develop the solution myself, I search for videos on the Internet and reflect on how I can decode the video into something I can do. In the clinic, I prepare by going through the procedure, imagining, and repeating each step in my head. I have seen well-performed procedures, so I know what it is supposed to look like. I adopt what I have seen others do and compare it to my previous impressions of surgeons doing something elegant *Constructed from 6 different informants’ expressions*
**Kinaesthetic**	I need to feel the training. It is a bodily learning experience in some way. It is not helpful to see others do it—I have to learn it through my arms. A good performance flows—it is not staccato. I have experienced that my hands can suddenly do things in the OR that they could not do before. It is not like, ‘now I use my left hand and now use my right’—it just happens naturally. My movements become fluid and intuitive. It is almost like skiing—that kind of bodily feeling *Constructed from 4 different informants’ expressions*
**Dialogue**	The way I like to do it with my supervisor is to say everything I plan to do and why I plan to do it before I do it. If I have no idea how to proceed, I either signal it non-verbally or ask directly. I often talk and think out loud—articulate when I do not know the next step. I prefer to put it into words so I do not feel that there is something unsaid. The more I can talk my way through it, the easier it is for me *Constructed from 4 different informants’ expressions*

**Table 4:** Text condensates for subgroups within the theme ‘transferable skills’

**Subgroup**	**Text condensate**
**Ambidexterity** **and coordination**	I have become more aware of using both hands, which is essential when performing procedures where you have to use your non-dominant hand to manoeuvre the tissue, keep tension, dissect and cut. It is a bimanual task with a bit of dancing between the two hands. The training program has helped me a lot; I have become more adept at using my non-dominant hand, which otherwise could be a bit sluggish. Before, it was like, ‘now I move my right hand, and now I move my left hand’, but now it is more fluid. My hands work more synchronously, and it comes more naturally now *Constructed from 7 different informants’ expressions*
**Instrument handling**	It has become easier to use the instruments and handle them properly. I no longer have to focus on the instruments and how I use my hands. I can handle the instruments correctly and directly grasp the structures my supervisor tells me to. It is the basics of instrument handling—grasping the tissue and knowing when and how to use the different instruments. The training program allowed me plenty of time with the instruments in my hands—I have to learn the rest through hours of sweating in the OR. If you know the instruments and how to handle them, both the supervisor and scrub nurses will notice it *Constructed from 8 different informants’ expressions*
**Visuospatial ability**	Grabbing a pen on the table is easy because we have a clear sense of where it is. But you do not have that sense at the beginning with laparoscopy. The sense of space and depth is entirely the same whether you practice laparoscopy in a shoebox or the OR. My visuospatial ability—looking at a screen and translating a 2D image into working on a 3D structure—has improved, which transfers directly into my clinical performance. The sense that if I move my instruments in this plane, it matches where I want to go on the screen. In the OR, if you try to grasp a structure and miss it, you risk losing your chance. You have to be very targeted and precise in your movements. I can apply my improved eye-hand coordination to endoscopy in general *Constructed from 6 different informants’ expressions*
**Approach to learning**	The training program has been an eye-opener on how to break learning situations down into smaller chunks. I have become more aware of how to approach tasks in different ways and that there is not necessarily only one correct way. I have started to use the training facilities in our department from time to time. The training program has given me instructions and provided me with extra motivation to train and become better step-by-step. I have learned that I can improve my skills if I want to and invest my time in training. The training has made me more confident in myself. I am less reluctant to state that I am ready for the next step in the OR *Constructed from 6 different informants’ expressions*

**Table 5:** Text condensates for subgroups within the theme ‘Transfer opportunities’

**Subgroup**	**Text condensate**
**Creating opportunities**	In the clinic, it is necessary to reach out. I always go through the programs to see if there are any procedures I can attend. I have pointed out to my supervisors that there are some procedures that I have to attend to get my training approved. I have become better at squeezing in and saying, ‘I want to suture’ or ‘I want to place the trocars.’ I do not control which and how many procedures I get to attend, but I try to influence what I am allowed to do when attending. You have to be skilled. You have to make yourself visible in everything you do. And you have to show them that you really want it. Otherwise, you will not get it. I have heard many times that ‘education is something you take. It is not something you get’. That is the training culture we have been taught *Constructed from 5 different informants’ expressions*
**Relations**	It is crucial who my supervisor is. Depending on whom I ask, I am sometimes allowed to perform, and sometimes I have to wait. It depends on their mood and whether our expectations are aligned. If the supervisor is stressed and impatient, he will quickly take over. It is difficult to navigate, but of course, you start to know the supervisors. I ask my peers, ‘How do you approach X?’ and ‘How do you get the opportunity to perform?’ A good supervisor is someone who does not intervene too soon and who gives hints rather than instructions. I prefer having a supervisor who I know and who has confidence in me. That way, I feel secure with the person. A new supervisor needs to know ​​who I am and what I can do. In the clinical setting, it does not matter if I pass the course with two A’s. What counts is how well-liked I am as a colleague, how strong I am in my theoretical knowledge, and how many procedures I have performed *Constructed from 8 different informants’ expressions*
**Training as** **‘Entry ticket’**	The training program provides a safe learning environment where I can train the things that I need to master before performing something more complex in the OR. The program provides me with the argument that ‘I have been training—I know how to do it, and I want to do it.’ Having received a certificate stating that I have obtained the basic skills level, I can say, ‘I have been on this course, and now I want to use my hands’. It is an entry ticket to the fun stuff. I use it as an argument to be allowed to do more things in the OR. I say, ‘by the way, I attended this training program 14 days ago’. Then they know that I have invested my time in training and that I really want it. It gives me a better chance for opportunities to perform in the OR *Constructed from 5 different informants’ expressions*
**Work planning**	None of my colleagues knows that I have been attending the training program, apart from maybe the work scheduler and chief physician, who have approved my leave of absence. It is not taken into account in the work planning in any way—that I should be prioritised for attending in the OR after the training program. Nothing changes in what I am doing in the clinic when I return from a training program. It is difficult because we are many trainees competing for few procedures. Everyone wants to be scheduled for the OR, but there are always changes to the plan. One sick leave, and then you are back in the outpatient clinic or doing rounds again. It is a bit absurd and somewhat inopportune. I wish the training was structured more in blocks. I guess no one thinks it is a bad idea, but it is difficult to turn into reality—they say *Constructed from 5 different informants’ expressions*

### Sportification of training

Early in the analysis, it became clear that many informants viewed and spoke of surgical training in a way reminiscent of a sports activity, focusing on competition, performance, strategy, and optimisation. Several informants stated that the laparoscopic training became ‘like a sport’ to them (Table 2). A preoccupation with a competitive element seemed to facilitate commitment to training both in the simulated and clinical environment. In the simulated environment, timing and grading of training tasks were driving factors of the competitive aspect. In both training environments, the competitive aspect of training was primarily reflected as competition with oneself focusing on improving own performances in training tasks and procedures. However, some informants also found competition in an unarticulated comparison with peers within the centralised and clinical training environments. In the clinical environment, some narratives reflected a focus on comparing numbers of procedures with peers, whereas in the simulated environment informants looked to peers to compare progression pace. The competitive aspects within the simulated environment were regarded mainly as motivating and fun, whereas narratives on competitive aspects in the clinical environment were more equivocal. Timing was a particularly competitive element in the simulated training setting because the two proficiency tasks (“Peg Transfer” and “Suture with Intracorporeal Knot”) adopted from the fundamentals of laparoscopic surgery [31] were rated as grade A or B according to set time limits. While grade B was sufficient to pass the program, informants expressed a strong desire to achieve grade A to obtain a certificate with proof of excellence. Stories of trying to optimise and develop strategies for each step in the training tasks to improve speed were prevalent. For instance, an informant recalled, “*Looking at those 48 seconds* (the grade A time limit), *you can only hold them* (the pegs) *for a maximum of four seconds if you want to get there. So, I try to count, so I know where I am. Then I do not have to look at that stupid clock that is counting too fast”* (I-2). Likewise, in the clinical setting, narratives reflected a focus on optimising performance and time for each step in the procedures.

Generally, a step-by-step approach and strategy for improving performance and attaining proficiency in the training tasks were apparent. Informants’ narratives from the simulated setting largely resembled their accounts from the clinical setting, where they also divided procedures into sub-steps that they trained and strove to master. The step-by-step process recurred in informants’ descriptions of excellent, fluent surgical performances: *“When I master each step and the transitions between the steps run smoothly, that is when I perform well. That is what I call flow”* (I-7).

### Modes of orientation

The text condensates in Table 3 provide insights into how informants expressed a visual orientation using actual visual inputs and mental visualisations in their planning, self-instruction and self-evaluation of training. The simulation-based training was influenced by informants’ visualisation of task performances assisted by viewing instructional videos and task demonstrations by instructors. Some sought additional instructions by searching the Internet for videos*.* A visual orientation was likewise present in the clinical setting, with informants using videos and inner renderings of excellent surgical performances to prepare for procedures. For example, one informant stated, *“I have seen well-performed procedures, so I know what it is supposed to look like” *(I-4).

Kinaesthetic learning experiences were prevalent. Informants described learning through the body, making sense of haptic input and noticing bodily movement and signals as part of their learning processes. Importantly, given the varied discourses, these findings should not be misinterpreted as an argument that the informants favoured a kinaesthetic learning approach or that it equated with broader embodied learning perspectives [32]. However, ‘learning by doing’ and experiences of internalising motor skills ‘through the body’ were salient in informants’ narratives in both training environments. For example, one informant recalled, “*One or two days after* (training), *I was on call, and I experienced that my hands could suddenly do things* (in the OR) *that they could not do before”* (I-3).

Informants described eliciting dialogues during their training. While informants in the centralised group could turn to the instructors for external dialogue during their training, the remote training group elicited internal dialogues for self-instruction. As one informant explained, “*If I do not succeed, I stop and ask myself: ‘What can you do differently to make it easier and do it faster next time?’”* (I-7)*.* Narratives also illustrated how informants preferred their clinical supervisors to engage in explicit, instructional and reassuring dialogues in the OR: *“The way I like to do it with my supervisor is to say everything I plan to do and why I plan to do it before I do it”* (I-11). Moreover, informants expressed uncertainty about the unspoken: *“One can get the feeling that something is unspoken ‘in the air’—that the supervisor will take over any minute because he is annoyed that nothing is happening. If I get that feeling, it is not constructive for my progress”* (I-9).

To summarise, two main themes, sportification of training (i.e., competition, timing and step-by-step approach) and different modes of orientation (i.e., visual, kinaesthetic and dialogue) characterised informants’ narratives of being engaged in training. We found that these characteristics were present in both the simulated and the clinical training environment. Interpreted within the theoretical framework of SRL, these findings uncover characteristics of self-regulatory subprocesses, such as task analysis, self-control, self-observation and self-judgment within the cyclical process of self-regulated surgical training.

### Transferable skills

A *transferable skill* is a general term for a skill that can be applied in different contexts. While many narratives centred around specific skills from the simulation-based training that trainees considered applicable in the clinical setting, others reflected how the simulation-based training influenced more generic skills, such as learning approach and training mindset.

As illustrated in Table 4, informants experienced improvements in ambidexterity and bimanual coordination in the OR due to training. In addition, several informants described improvements in their instrument handling and a general increase in their awareness of instrument selection. Interestingly, informants linked these skills to recognition in the OR. As expressed by one of the informants, *“The nurses keep an eye on you. The more confident you are, the more they are on your side. If you handle things properly and know the name of the instruments, then they will hand them to you in a good way”* (I-3).

Another important transfer outcome of the training program was improved visuospatial ability and hand-eye coordination. Informants referred to immediate improvements in these skills when attending laparoscopic procedures. Moreover, some informants experienced improvements in their ability to perform other endoscopic procedures (cystoscopies, colonoscopies, etc.). In addition to these noticeable and applicable improvements in skills, the training program triggered informants’ awareness of learning strategies and general approaches to training. For instance, one informant said, *“I think it* (the training program) *has been an eye-opener on how to break learning situations down into smaller chunks”* (I-11). Furthermore, the training program influenced informants’ training mindset by providing insight into their training potential. Awareness of their own skills and potential contributed to increased confidence, motivation and recognition of skills training as essential to obtaining and maintaining competencies as a surgeon.

### Transfer opportunities

The informants were very preoccupied and reflective about their opportunities to perform in the clinical setting. Viewing the training program as an entry ticket, the informants regarded it as a useful argument for being allowed to perform in the OR: *“Now I can say ‘I have been on this course, now I want to use my hands.’ As I said, it is an entry ticket to the fun stuff”* (I-3).

Remarkably, at the same time, informants mentioned a lack of attention and recognition of their participation in the training program in their clinical work environment and were convinced that no supervisor would ever request their course diploma or bother about their training grades. Some informants described referring and hinting to their clinical supervisors about their course participation to argue for more procedural involvement. The informants had a general perception that creating opportunities to perform in the OR requires outreach and being visible. An informant explained, *“You have to be skilled. You have to make yourself visible in everything you do. And you have to show them that you really want it. Otherwise, you will not get it. I have heard many times that ‘education is something you take. It is not something you get’” *(I-11).

As illustrated in Table 5, the relational aspect was prominent in informants’ narratives on transfer of training. In particular, informants perceived their relationship with the clinical supervisor as decisive for their opportunities to perform in the OR. Difficulties in decoding this relational aspect were prominent, and informants described different strategies for navigating day-to-day interactions with their supervisors, trying to create opportunities to perform by obtaining mutual confidence and respect. Strategies ranged from purposefully seeking out well-known supervisors to asking peers for advice on how to approach certain supervisors.

Furthermore, organisational factors such as work planning and priorities were described as typical barriers to the transfer of training. For instance, an informant stated, *“It is not taken into account in the work planning in any way—that I should be prioritised for attending in the OR after the training program”* (I-5). Informants were frustrated about this lack of coherence between the training course and the clinical work and saw much potential for increased transfer outcomes in optimising this aspect.

## Discussion

Surgery and skills training has previously been compared to other high-performance domains [33, 34]. This study substantiates this comparison by finding that trainees’ narratives on engaging in surgical training were reminiscent of accounts from engaging in a sports activity. Sportification can have two separate meanings. Firstly, it can refer to applying sports elements (e.g., rules, training regimes, performance standards, time regulations) in a non-sport activity. Secondly, it can refer to viewing, organising and regulating a non-sport activity in a way that resembles sports [35]. In the present training programme, elements and structures (e.g., grades, timing, proficiency levels) resembled sports elements. We found that the informants generally viewed, regulated and engaged in surgical training in a manner reminiscent of a sports activity (i.e., the second definition of sportification). They embraced competition and comparison of performances with themselves and others as an inevitable training regime. Furthermore, they accepted the external regulation of rules, exercises and proficiency criteria that rewarded or possibly induced a competitive mindset within the SBST program. These findings are perhaps not surprising since sports play a prominent role in popular culture and society in general [36, 37].

According to Zimmerman (2000), SRL involves three cyclical phases: forethought, performance and a self-reflection phase [15]. Our findings demonstrate characteristics of how trainees modulated and adapted their learning approaches during training. Trainees did this by sportification and by the presented different modes of orientation in training. We argue that the scaffolding of the PBT program supported trainees’ goal orientation and self-regulatory training processes. Written and visual instructions provided stepwise process goals that supported trainees’ self-instruction and visual orientation (imagery) in the *performance phase*. Our findings also suggest that further application of kinaesthetic learning principles may contribute to this phase, which is supported by a previous study [38] and aligns with established theories of skills acquisition [7, 39]. Opportunities for video reviewing and written summary feedback from instructors assisted trainees in the *self-reflection* and *forethought phases*. Correspondingly, a recent metanalysis advocates applying sport psychology techniques to improve clinical skills of health professionals [40]. Interestingly, we found that the self-regulatory learning processes in both the simulated and the clinical environment mirrored some of these sports psychology techniques, namely imagery, self-talk and goal setting. These findings raise questions about how instructional designers and clinical and educational coordinators may support and benefit from trainees’ self-regulatory processes in ways that transcend training environments. Future research should explore how simulation-based training can support ongoing SRL in clinical practice and, in turn, investigate how that affects the transfer of training.

Although the primary purpose of this exploratory study was not comparative, we did notice slight differences between the two different training groups. While the remote training group only had themselves to compete with, some informants from the centralised training group also mentioned a competitive orientation towards peers. The same was true for the orientation towards dialogues during training; the remote training group elicited an inner dialogue, while the centralised group relied more on conversations with the instructors who were present. These findings add to research suggesting that trainees in a proctored and grouped training setting somewhat rely on observing and mirroring peer performances and receiving concurrent feedback from instructors [14]. Similarly, we found that trainees engaging in remote laparoscopic training also viewed and regulated their training in ways resembling those of a sports activity. Despite being unable to interact with instructors and peers concurrently, they used similar modes of orientation in their training. These findings add to research that investigated the effect of different types of instructor feedback and the value of video instructions in basic surgical skills training [41–43].

Trainees had ambiguous perceptions and experiences of transfer of training to the clinical environment. On the one hand, they reported applicable performance improvements from training and viewed the program as an ‘entry ticket’ to the OR. On the other hand, they struggled for opportunities to transfer and expressed difficulties navigating the relational aspects and overcoming organisational barriers for transfer of training. Previous SBST studies have primarily focused on training design and on establishing evidence for transfer of training by demonstrating performance improvements in the OR after training [2]. As mentioned above, established models of the transfer process emphasise that the actual conditions of transfer are influenced by two other main factors, namely trainee characteristics and the work environment [4–6]. Even though an investigation of trainee characteristics (e.g., cognitive ability and motivation) from a psychological perspective was outside the scope of this study, we generally found the first-year trainees to be adept and highly motivated individuals. Evidence from various training domains emphasise that transfer climate, support, opportunity to perform and post-training follow-up in the work environment critically influence the conditions of transfer [4–6]. We found that trainees did not experience adequate support and recognition of their participation in SBST in the clinical work environment. In transitioning from the simulation-based training setting to the clinical setting, trainees experienced lack of timely opportunities to intensify relevant surgical activities, partly due to what they experienced to be inadequate work planning. The findings of a previous study by our research group support the idea that these experiences reflect reality [44]. Further studies that focus on the influence of the clinical work environment on the transfer of training process are recommended.

### Strengths and limitations

This study contributes to existing knowledge of surgical training by providing insights into the more specific characteristic processes of surgical training engagement and transfer of training. Our study was informed by well-established theories on SRL and transfer of training. This theory-informed research approach allowed us to draw from existing knowledge from the outset of the study while at the same time using the theories as lenses for analysing and interpreting ﻿new insights from our specific subject field based on narrative data [45]. On the other hand, a robust theoretical outset may limit the researchers’ receptiveness to other perspectives. We tried to overcome this limitation by bracketing our preconceptions and undertaking a collaborative approach to the analysis [29].

By conducting two interviews separated in time, we allowed informants to develop and elaborate on their experiences, contributing to an in-depth understanding of the subject matter. Sharing sample population with a comparative study harbours the risk that this would divert focus from our intended research objectives. However, the present study served a different purpose, and research questions and methods differed substantially from our previous work [24]. We did not find that the informants’ narratives reflected a substantial focus on allocated training groups or the derived consequences thereof. However, we might arguably have identified more differences if a further comparison of the two training modalities had been the focus of the present study.

The ﻿extent to which the findings and conclusions drawn from this type of study applies to other ﻿contexts is always disputable. The purpose of this exploratory study was to contribute to further understanding SRL processes and perspectives of transfer in the transition between simulation-based and clinical surgical training. As such, the findings may have utility across contexts, where similar training situations and transitions exist.

## Conclusions

In developing postgraduate training initiatives, instructional designers should consider how to support learning processes and the transfer of training into clinical practice. Knowledge of educational supports and factors influencing the transfer process is essential in this endeavour and understanding trainees’ perspectives is key. Further studies that address the supervisor and organisational perspectives are warranted.

The insights from our study suggest that instructional designers may leverage the sportification of training in motivating engagement and supporting trainees in self-regulatory training processes that transcend training environments. In addition, our findings substantiate how educational supports affect trainees’ self-regulatory learning processes.

We have elaborated on the complexity of the transfer process in finding that trainees struggle in aligning their expectations with the actual clinical opportunities and in navigating relational and organisational transfer aspects in the clinical work environment. Accordingly, we argue for adopting a more holistic approach to the transfer of surgical training that focuses not solely on training design and methods but acknowledges that transfer occurs before, during and after training and should be supported at every stage. In that endeavour, instructional designers should collaborate closely with clinical training coordinators to ensure that supportive measures for transfer are in place in both training environments and to better align training opportunities with clinical work planning.

## Data Availability

The datasets used and analysed during the current study are not publicly available due to the relatively small number of participants and the possibility of compromising anonymity/individual privacy; however, data may be made available from the corresponding author at a reasonable request.
